# Acute mesenteric ischemia and outcomes in ICU survivors

**DOI:** 10.1186/2197-425X-3-S1-A537

**Published:** 2015-10-01

**Authors:** SL Gans, FK Gibbons, MA Boermeester, KB Christopher

**Affiliations:** Surgery Department, Academic Medical Centre, Amsterdam, the Netherlands; Massachusetts General Hospital, Pulmonary and Critical Care Medicine, Boston, MA USA; Renal Division, Brigham and Women's Hospital, Boston, MA USA

## Introduction

Acute Mesenteric Ischemia is associated with substantial in-hospital mortality. It is not known if Acute Mesenteric Ischemia is risk factor for outcomes in ICU survivors.

## Objectives

We hypothesized that ICU patients with Acute Mesenteric Ischemia who survive hospitalization would be at high risk for adverse events following discharge.

## Methods

We performed a two center observational study of patients treated in medical and surgical intensive care units in Boston, Massachusetts. We studied 82,583 patients, aged ≥ 18 years, who received critical care between 1997 and 2012 and survived to hospital discharge. The exposure of interest was Acute Mesenteric Ischemia (AMI) defined as an ICD-9 code of vascular insufficiency of intestine (ICD-9 557) during the hospitalization. The primary outcome was 30-day post-discharge mortality determined by the US Social Security Death Master File. Secondary outcome was unplanned 30 day hospital readmission. Adjusted odds ratios were estimated by multivariable logistic regression models with inclusion of terms for gender and the Acute Organ Failure score (1), a validated ICU risk-prediction score inclusive of terms for age, race, comorbidity, patient type (surgical vs medical), sepsis and acute organ failure. Mortality was also analyzed with a risk-adjusted Cox proportional hazards regression model.

## Results

In the cohort of ICU survivors (N = 82,583) 58% were men, 80% were white, 51% were surgical patients, 10% had sepsis, 2% had bowel surgery and the mean age was 61 years. 30-day post-discharge mortality was 3.8% and 30-day hospital readmission was 14.1%. In ICU survivors with AMI (N = 644), 47.4% had bowel surgery, the 30-day post-discharge mortality rate was 7.3%, and the 30-day readmission rate was 21.3%. AMI was a robust predictor of post-discharge mortality. The adjusted odds of 30-day post-discharge mortality in patients with AMI was 1.62 (95%CI 1.19-2.20), relative to patients without AMI. Further, the adjusted odds of 30-day readmission in patients with AMI was 1.56 (95%CI 1.29-1.89), relative to patients without AMI. Patients with AMI experienced a significantly higher risk of death following discharge during 269 person-years of follow-up than patients without AMI [HR 1.16 (95% CI, 1.01-1.33)]. Finally, in a propensity score matched analysis of 642 patients with AMI and 642 patients without AMI, the odds of 30-day post-discharge mortality in patients with AMI was 1.67 (95%CI 1.04-2.69), relative to patients without AMI.

## Conclusions

Acute Mesenteric Ischemia patients are among a high-risk group of critically ill survivors. Acute Mesenteric Ischemia is a robust predictor of post hospital discharge mortality and hospital readmission in survivors of critical care.
